# Fatty liver index and risk of type 2 diabetes of adults with normoglycemia: Insights into insulin sensitivity and beta-cell function

**DOI:** 10.1371/journal.pone.0327058

**Published:** 2025-06-26

**Authors:** Ji Hyun Bae, Min Jin Lee, Su Hyun Kim, Joo Yeon Kim, Ah Reum Khang, Yang Ho Kang, Dongwon Yi

**Affiliations:** 1 Division of Endocrinology and Metabolism, Department of Internal Medicine, Pusan National University Yangsan Hospital, Pusan National University School of Medicine, Yangsan, South Korea; 2 Research Institute for Convergence of Biomedical Science and Technology, Pusan National University Yangsan Hospital, Yangsan, South Korea; Johns Hopkins University Bloomberg School of Public Health, UNITED STATES OF AMERICA

## Abstract

The fatty liver index (FLI) is a simple tool used to assess metabolic dysfunction-associated fatty liver disease (MAFLD). Previous studies have shown the utility of the FLI as an early predictor of diabetes for patients with prediabetes. We evaluated whether the FLI could predict the development of type 2 diabetes mellitus (T2DM) with normal glucose tolerance (NGT) by performing a retrospective assessment of a community-based cohort over the course of 18 years. We analyzed data of 6,083 adults with NGT available from the Korean Genome and Epidemiology Study database. Participants were stratified into the following three groups based on the FLI: low, FLI < 30; intermediate, FLI 30–59; and high, FLI ≥ 60. Cox proportional hazards regression models evaluated the T2DM risk differences. Insulin sensitivity and secretion markers were compared using multivariate linear regression and an analysis of covariance. The predictability of the FLI for T2DM was analyzed by comparing the area under the receiver-operator characteristic (ROC) curve (AUC) values from the ROC analysis. The cumulative incidence of T2DM was 31.9% for the high FLI group; however, it was 11.3% for the low FLI group (log-rank test, P < 0.0001). For individuals with NGT, a high FLI was associated with an increased T2DM risk (hazard ratio [HR], 3.42; 95% confidence interval [CI], 2.91–4.00). After adjusting for insulin sensitivity and secretion markers, FLI remained an independent predictor of T2DM (HR, 1.96, 95% CI, 1.54–2.50). The homeostasis model assessment of insulin resistance results and composite insulin sensitivity index of the high FLI group were higher than those of the other groups (P < 0.0001). However, the disposition index and insulin secretion-sensitivity index-2 of the high FLI group were lower than those of the intermediate FLI group (P = 0.027 and P = 0.011, respectively). The ROC analysis confirmed that the FLI had the highest predictive ability for T2DM (AUC, 0.654; P < 0.05) development in individuals with NGT compared to other insulin sensitivity and secretion markers. The FLI is an early predictor of T2DM that reflects underlying insulin sensitivity and β-cell function. These findings underscore the role of liver steatosis in the early T2DM pathogenesis and highlight the need for early preventive lifestyle interventions among individuals with normoglycemia and high FLI values.

## Introduction

Type 2 diabetes mellitus (T2DM) is a leading cause of death associated with cardiovascular disease and a chronic progressive disease accompanied by various complications. Therefore, it creates a burden on individuals and society [1–3]. In 2021, the 10th edition of the International Diabetes Federation Diabetes Atlas reported that 537 million adults 20–79 years of age worldwide have diabetes, and this number is projected to increase to 783 million by 2045 [4]. According to the diabetic fact sheets in Korea, the prevalence of diabetes among individuals 30 years or older increased from 4.8 million (13.7%) in 2016 to 5.3 million (15.5%) in 2024 [5]. Hepatic regulation is crucial to glucose metabolism. Hepatic steatosis leads to hepatic insulin resistance, inappropriate endogenous glucose production, and abnormal lipid metabolism, resulting in T2DM [6,7]. The bidirectional association between nonalcoholic fatty liver disease (NAFLD) and T2DM is well-established, although its pathophysiology remains unclear. According to a large-scale meta-analysis, patients with NAFLD had a 2.22-times higher risk of developing T2DM compared to that of those without NAFLD. Furthermore, this risk can increase up to 4.74 times for individuals with severe NAFLD [8]. Recently, a shift from classifying liver steatosis as NAFLD based on its developmental pathway to classifying it as metabolic dysfunction-associated fatty liver disease (MAFLD) and steatotic liver disease has occurred. This new classification system focuses on the presence of liver steatosis accompanied by metabolic abnormalities, regardless of the underlying cause [9]. The MAFLD criteria include T2DM, obesity, and metabolic risk abnormalities (including the insulin resistance score), thus emphasizing a strong association with metabolic disorders [9]. Liver biopsy is the most reliable method of diagnosing steatotic liver disease. However, liver biopsy is not suitable for screening patients with mild fatty liver and is associated with a high cost and the risk of complications. Although abdominal ultrasonography is relatively accurate and useful, it is not suitable for repeated use because of its high cost. The fatty liver index (FLI) is as effective as ultrasonography for predicting fatty liver disease [10,11]. The FLI, which is based on a robust indicator of MAFLD pathophysiology, is a simple tool that can be used to assess MAFLD [12,13] and metabolic syndromes [14,15]. Previous studies have demonstrated the utility of the FLI as an early predictor of T2DM development in patients with prediabetes [16,17]. However, limited assessments of its effectiveness for individuals with normal glucose tolerance (NGT) have been conducted. Therefore, we aimed to assess whether the FLI could effectively reflect metabolic dysfunction and serve as an early predictor of T2DM, even for individuals with NGT, by performing a longitudinal assessment of a community-based cohort over the course of an 18-year period. Additionally, we compared the differences in insulin sensitivity and insulin secretion markers according to the FLI and investigated their correlation with the FLI. We also evaluated the predictive ability of the FLI relative to these markers for individuals with or without NGT.

## Materials and methods

### Study population

During this study, we analyzed data from the Ansan and Ansung cohort, which is a subset of the Korean Genome and Epidemiology Study (KoGES), which is a longitudinal, prospective cohort study conducted by the Korean Centers for Disease Control and Prevention (KCDC), to investigate the genetic and environmental determinants of chronic diseases. The KoGES is a large-scale prospective study of 10,030 Korean individuals 40–69 years of age that was initiated in 2001; participants were recruited based on age, sex, and residential location from the urban area of Ansan using a random sampling method and from the rural area of Ansung using a cluster sampling method. Baseline data collection was conducted from 2001 to 2002. Thereafter, continuous biennial follow-up assessments were performed. Participants underwent standardized assessments every 2 years that included anthropometric measurements, laboratory tests, and structured questionnaires [18]. A 75-g oral glucose tolerance test was also conducted biennially to reassess the glycemic status until a clinical diagnosis of diabetes was determined. We obtained and retrospectively analyzed the baseline survey data (2001–2002) and follow-up data (2019–2020) from the National Institute of Health of Korea and Korea Disease Control and Prevention Agency on November 23, 2023. The KoGES provides only de-identified data in compliance with the Personal Information Protection Act and the Statistics Act, thus ensuring that individuals cannot be identified from the survey data. Therefore, we only had access to anonymized data that did not contain any personally identifiable information during or after data collection.

**Fig 1:**
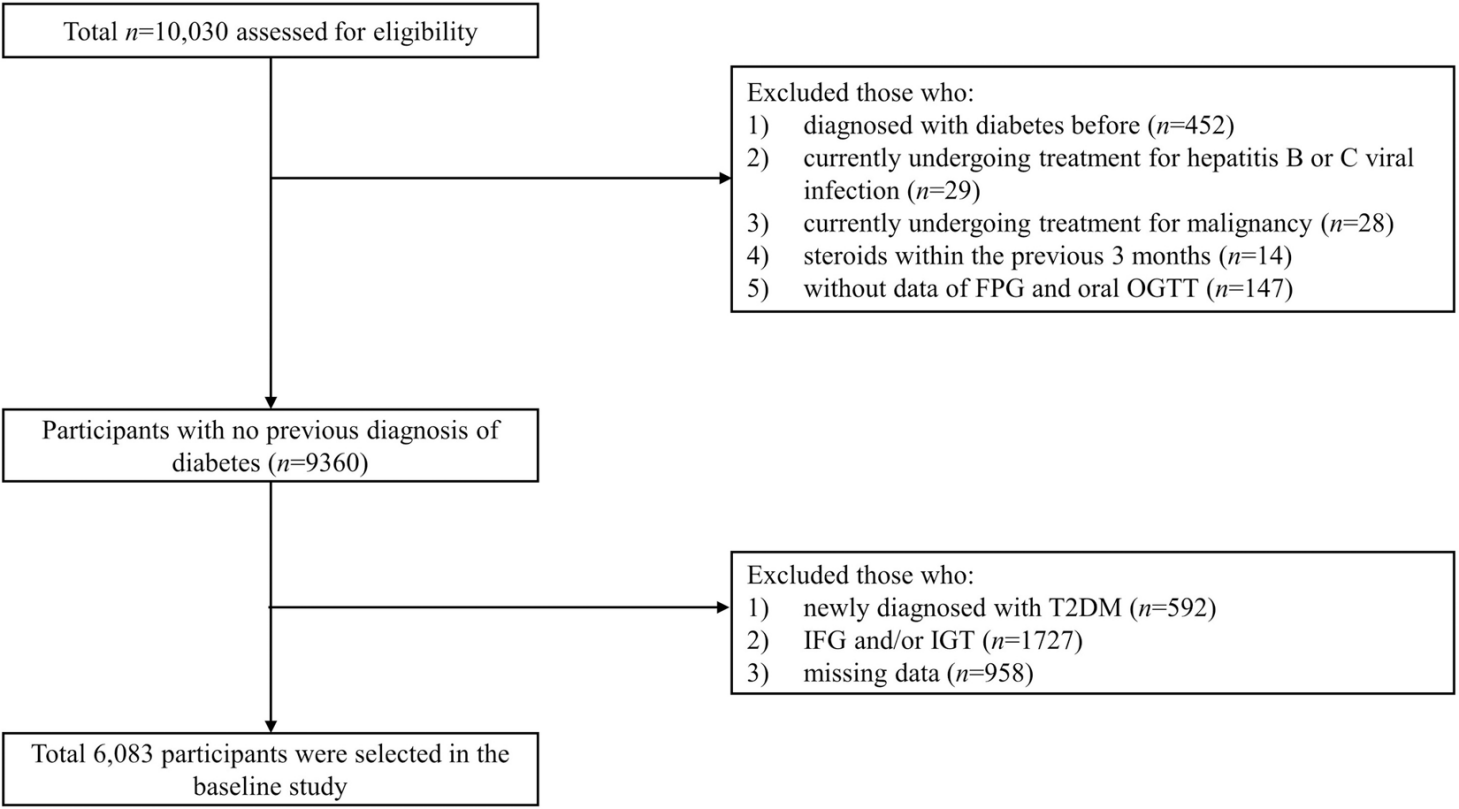
Flowchart of the study population. Abbreviations: FPG, fasting plasma glucose; IFG, impaired fasting glucose; IGT, impaired glucose tolerance; OGTT, oral glucose tolerance test; T2DM, type 2 diabetes mellitus.

From the 10,030 participants at baseline, we excluded individuals with preexisting diabetes, those undergoing treatment for malignancy or viral hepatitis (hepatitis B or C), those who were treated with corticosteroids in the past 3 months, and those with missing essential data regarding fasting plasma glucose (FPG) and the oral glucose tolerance test (OGTT). Additionally, participants who were newly diagnosed with T2DM at baseline, those with impaired fasting glucose (IFG) and/or impaired glucose tolerance (IGT), and those with other missing data were excluded. After applying these criteria, 6,083 participants were included in the final analysis (Fig 1). Ethical approval of the study protocol was obtained from the Korean Centers for Disease Control and Prevention Ethics Committee, and written informed consent was obtained from all participants. This study adhered to the principles of the Declaration of Helsinki and was approved by the Institutional Review Board of Pusan National University Yangsan Hospital (IRB no. 55-2023-028).

### Assessment of anthropometric and biochemical parameters

The participants visited designated community clinics where trained healthcare professionals conducted anthropometric measurements and biochemical tests. The body mass index (BMI) was calculated by dividing the weight (kg) by the square of the height (m^2^). Waist circumference (WC) was measured midway between the lowest rib margin and the iliac crest during exhalation. Blood pressure (BP) was measured three times on the right upper arm using a mercury sphygmomanometer, and the average of the second and third readings was recorded. Smoking history was categorized as never smoker (fewer than 100 cigarettes during the lifetime of the individuals), ex-smoker (at least 100 cigarettes during the lifetime of the individual but not currently smoking), or current smoker (at least 100 cigarettes during the lifetime of the individual and currently smoking). Alcohol consumption data were gathered during the interviews and quantified as grams per week.

Hypertension was defined as systolic BP (SBP) ≥140 mmHg, diastolic BP (DBP) ≥90 mmHg, or the use of antihypertensive medications. A family history of diabetes was noted if first-degree relatives were involved. Biochemical testing required at least 8 h of fasting and included FPG, insulin, total cholesterol (TC), high-density lipoprotein cholesterol (HDL-C), and triglyceride (TG) levels. This cohort did not include direct measurements of low-density lipoprotein cholesterol (LDL-C). Therefore, LDL-C levels were calculated using the Friedewald formula [19]: LDL-C = TC − (TG/5) − HDL-C, excluding cases with TG > 400 mg/dL (112 individuals). Plasma samples for glucose and insulin were collected at 0, 60, and 120 min during a 2-h 75-g OGTT. Plasma glucose levels were measured using the hexokinase method, whereas insulin levels were determined using a radioimmunoassay.

### Definition and outcomes

NGT was defined as an FPG level <100 mg/dL and 2-h plasma glucose level <140 mg/dL during the OGTT; this definition excluded individuals diagnosed with T2DM. T2DM was diagnosed based on any of the following criteria: FPG ≥ 126 mg/dL; 2-h plasma glucose ≥200 mg/dL during the OGTT; or the use of antidiabetic medications, including insulin. HbA1c (glycated hemoglobin) levels ≥ 6.5% were not included in the diagnostic criteria because the study was conducted before the American Diabetes Association introduced HbA1c as a standard diagnostic tool in 2010. The primary outcome of this study was the incidence of T2DM during the 18-year follow-up period.

For individuals with NGT, we calculated the FLI based on initial data from 2001. The variables included in the FLI calculation were the TG level, BMI, gamma-glutamyl transferase (GGT) level, and WC. The FLI ranged between 0 and 100.


$$\begin{array}{cc} FLI\;= & \;(e^{0.953\times loge(TG)+0.139\times BMI+0.718\times loge(GGT)+0.053\times WC-15.745})/\\ & (1\;+\;e^{0.953\times loge(TG)+0.139\times BMI+0.718\times loge(GGT)+0.053\times WC-15.745})\;\times \;100 \end{array}$$

The FLI values were categorized as follows and as previously described [20]: FLI < 30, low; FLI 30–59, intermediate; and FLI ≥ 60, high. A low FLI indicated a very low likelihood of fatty liver (negative likelihood ratio = 0.2), whereas a high FLI was strongly associated with a high probability of fatty liver (positive likelihood ratio = 4.3) [20]. Insulin resistance (homeostasis model assessment [HOMA] of insulin resistance [HOMA-IR]) was calculated using the following formula: HOMA-IR = fasting plasma insulin (FPI) (mmol/L) × FPG (μU/mL)/ 22.5. Pancreatic β-cell function (HOMA of pancreatic β-cell function) was calculated using the following formula: HOMA of pancreatic β-cell function = [FPI (μU/mL) × 20]/ [FPG (mmol/L) − 3.5] [21]. Markers derived from the OGTT to evaluate insulin sensitivity and secretion were calculated as follows: composite or Matsuda insulin sensitivity index (ISI): ISI = 10,000/ √(FPG × FPI × mean plasma glucose × mean plasma insulin) [22]; 60-min insulinogenic index (IGI_60_): IGI_60_ = (plasma insulin at 60 min – FPI)/ (plasma glucose at 60 min − FPG) [23]; and oral disposition index_60_: disposition index = IGI_60_ × composite ISI [24]. The area under the receiver-operator characteristic (ROC) curve (AUC)_insulin/glucose_ was calculated as (AUC_insulin_)/ (AUC_glucose_) using the trapezoidal rule during the 75-g OGTT [25]. The insulin secretion-sensitivity index-2 (ISSI-2) was calculated as follows: ISSI-2 = AUC_insulin/glucose _× composite ISI [24].

### Statistical analysis

We compared the baseline characteristics of the study population across the FLI groups (low, intermediate, and high) using a one-way analysis of variance for continuous variables and a chi-squared test for categorical variables. Logarithmic or modified Box-Cox transformations were applied to normalize the skewed variables. Continuous data are expressed as means ± standard deviations or medians (25th and 75th percentiles), whereas categorical data are summarized as frequencies and percentages. A multivariate linear regression analysis was conducted to assess the independent association between the FLI as a continuous variable and insulin sensitivity and secretion markers in the baseline population. Model 1 comprised the correlation between the FLI and markers of insulin sensitivity and secretion. Model 2 comprised the beta coefficient of the FLI and P value after adjusting for age, SBP, DBP, TC, and HDL-C. Model 3 was adjusted for BMI to examine the association with each marker. Additionally, we used an analysis of covariance to evaluate the differences in insulin sensitivity and secretion markers across the three FLI groups. Covariates, including age, sex, SBP, BMI, TC, and HDL-C, which are established risk factors for T2DM, were adjusted [26].

The cumulative incidence of T2DM was illustrated using Kaplan–Meier curves and log-rank tests. Differences in the cumulative incidence across the FLI groups were assessed using log-rank tests. To determine the risk of T2DM while controlling for potential confounding factors, we used multivariate Cox proportional hazard regression models. Hazard ratios (HRs) for the T2DM incidence were reported with corresponding 95% confidence intervals (CIs). Model 1 evaluated the crude association between the T2DM risk and FLI. Model 2 adjusted for common T2DM risk factors, including age, sex, BMI, BP, lipid profile, smoking, alcohol consumption, and family history of diabetes [26]. Model 3 included additional adjustments for baseline insulin sensitivity and secretion markers to assess the independent effects of the FLI. The Schoenfeld residuals test showed that the proportional hazards assumption was satisfied in these models.

The ROC analysis was performed to calculate the AUC to evaluate the accuracy of the FLI, insulin sensitivity, and insulin secretion markers when predicting the T2DM incidence. The Youden index was calculated to improve diagnostic precision. Differences between the AUCs were examined using the DeLong test, and statistical significance was set at a two-tailed P < 0.05. All statistical analyses were performed using R software (version 4.2.3; R Foundation for Statistical Computing, Vienna, Austria).

## Results

### Baseline characteristics

The baseline characteristics of the 6,083 participants with NGT were categorized into three FLI groups (Table 1). In our study, we observed a distinct sex-based difference in the distribution of FLI categories. Among the 2,891 male participants, 530 (18.3%) were classified as the high FLI group (≥60), whereas only 200 (6.3%) of the 3,192 female participants were classified as the high FLI group. Participants in the high FLI group had higher mean BMI, WC, SBP, DBP, FPG, 1-h glucose, and 2-h glucose levels during the OGTT as well as higher mean HbA1c, TC, TG, AST, ALT, and GGT levels than those of participants in the low FLI group. Conversely, HDL-C levels were lower in the high FLI group. Although smoking status did not differ significantly among the groups, alcohol consumption (measured in grams of alcohol) was higher in the high FLI group. No significant difference in the family history of diabetes was observed. The HOMA-IR values increased and the composite ISI values decreased in the high FLI group. IGI_60_ and AUC_insulin/glucose_, which are insulin secretion markers, were increased in the high FLI group. The ISSI-2 decreased in the high FLI group, although the 60-min disposition index (DI_60_) showed no significant differences across the FLI groups.

**Table 1:** Baseline characteristics of the study population according to the fatty liver index (FLI).

Characteristic	Total	FLI			*P* value
		Low (<30)	Intermediate (30–59)	High (≥60)	
n	6083	3791	1562	730	
FLI^a^	21.3 (9.9,41.9)	12.1 (6.8,19.3)	42.1 (35.6,49.8)	71.1 (65.6,79.4)	<0.0001
Sex, n (%)					<0.0001
Male	2891	1455 (38)	906 (59)	530 (73)	
Female	3192	2336 (62)	656 (49)	200 (27)	
Age, year	51.3 ± 8.7	51.0 ± 8.8	52.1 ± 8.6	51.2 ± 8.2	<0.0001
BMI, kg/m^2^	24.3 ± 3.0	22.9 ± 2.4	25.9 ± 2.4	27.7 ± 2.9	<0.0001
WC, cm	81.8 ± 8.7	77.6 ± 6.8	87.1 ± 5.7	92.4 ± 7.0	<0.0001
SBP, mmHg	119.4 ± 17.5	116.5 ± 17.2	123.1 ± 16.8	126.5 ± 17.3	<0.0001
DBP, mmHg	79.4 ± 11.2	77.1 ± 10.8	82.4 ± 10.7	85.2 ± 11.2	<0.0001
Alcohol intake, g/week	0 (0,7.8)	0 (0,4.1)	0.7 (0,17.4)	8.1 (0,28.9)	<0.0001
Smoking, *n* (%)					<0.0001
Never smoker	3780 (62.9)	2630 (69.3)	847 (54.2)	303 (41.5)	
Ex-smoker	732 (12.2)	341 (9.0)	246 (15.7)	145 (19.9)	
Current smoker	1495 (24.9)	765 (20.2)	455 (29.1)	275 (37.7)	
Hypertension, *n* (%)	487 (8.0)	200 (5.3)	190 (12.2)	97(13.3)	<0.0001
Family history of diabetes, *n* (%)	571 (9.4)	354 (9.3)	147 (9.4)	70 (9.5)	0.977
FPG, mg/dL	81.2 ± 7.0	80.3 ± 6.8	82.2 ± 7.1	83.5 ± 7.0	<0.0001
1-hour glucose, mg/dL	131.3 ± 35.4	126.6 ± 35.4	136.0 ± 34.1	145.3 ± 33.2	<0.0001
2-hour glucose, mg/dL	102.5 ± 20.8	100.8 ± 20.8	104.1 ± 20.4	108.2 ± 20.1	<0.0001
HbA1c, %	5.50 ± 0.3	5.46 ± 0.3	5.56 ± 0.3	5.61 ± 0.3	<0.0001
TC, mg/dL	187.4 ± 33.4	182.4 ± 31.9	192.5 ± 33.0	202.2 ± 36.2	<0.0001
HDL-C, mg/dL	44.9 ± 10.0	46.9 ± 9.9	42.3 ± 9.2	40.6 ± 9.1	<0.0001
TG^a^, mg/dL	128 (95,176)	107 (85,136)	161 (128,209)	237 (179,312)	<0.0001
LDL-C, mg/dL	113.4 ± 30.7	112.4 ± 29.3	115.6 ± 31.8	114.1 ± 35.4	0.002
AST, IU/L	28.2 ± 12.1	26.3 ± 8.5	29.2 ± 21.8	35.9 ± 21.8	<0.0001
ALT, IU/L	25.7 ± 16.3	21.3 ± 10.5	29.0 ± 17.4	41.4 ± 25.0	<0.0001
GTP^a^, IU/L	17 (11,30)	13 (10,19)	25 (16,41)	50 (31,90)	<0.0001
Fasting insulin^a^, mIU/L	6.8 (5.1,9.4)	6.4 (4.9,8.5)	7.5 (5.5,10)	8.5 (6,11.3)	<0.0001
HOMA-IR^a^	1.4 (1.0,1.9)	1.3 (1.0,1.7)	1.5 (1.1,2.0)	1.7 (1.2,2.4)	<0.0001
HOMA-β^a^	142.1 (94.7,208.8)	139.8 (92.6,203)	142.8 (96,216)	153.6 (100.4,230.4)	0.002
Composite ISI^a^	10.1 (7.1,14.5)	11.1 (8.1,15.7)	8.9 (6.3,12.8)	7.5 (5.3,10.7)	<0.0001
IGI_60_^b^	6.6 (1.2,14.7)	5.9 (0.9,13.3)	7.9 (1.9,16.7)	8.2 (2.4,16.3)	<0.0001
Oral DI_60_^b^	62.7 (16.5,127.1)	62.8 (14.0,131.9)	66.5 (20.2,128.5)	55.2 (23.7,104.8)	0.056
AUC_insulin/glucose_^a^	3.3 (2.0,5.1)	3.0 (1.9,4.6)	3.7 (2.3,5.7)	4.1 (2.4,6.5)	<0.0001
ISSI-2^a^	32.4 (23.9,43.1)	33.1 (24.3,44)	32.2 (23.5,42.8)	29.5 (22.5,39.4)	<0.0001

Values are presented as mean ± standard error, median (interquartile range), or number (%).

^a^Logarithmic transformation was performed prior to the analysis.

^b^Modified Box-Cox transformation was applied to calculate P values.

Abbreviations: AUC, area under the curve; BMI, body mass index; DBP, diastolic blood pressure; DI, disposition index; FPG, fasting plasma glucose; HbA1c, glycated hemoglobin; HDL-C, high-density lipoprotein cholesterol; LDL-C, low-density lipoprotein cholesterol; HOMA-β, homeostasis model assessment of pancreatic β-cell function; HOMA-IR, homeostasis model assessment of insulin resistance; IGI, insulinogenic index; ISI, insulin sensitivity index; ISSI-2, insulin secretion-sensitivity index-2; SBP, systolic blood pressure; TC, total cholesterol; TG, triglycerides; WC, waist circumference.

### Association among the FLI and insulin sensitivity and secretion markers

**Fig 2:**
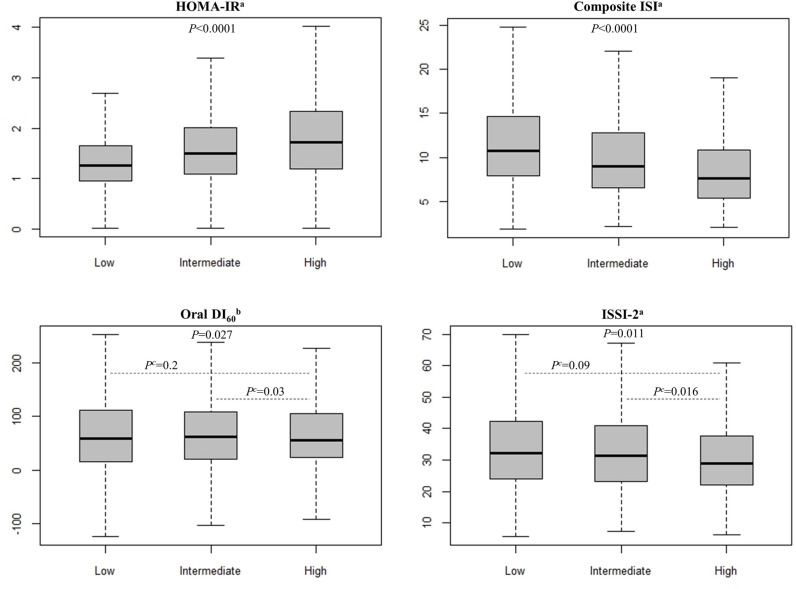
OGTT-derived insulin sensitivity and secretion markers across FLI groups adjusted for covariates. The *P* values at the top of the figures were calculated using an ANCOVA adjusted for age, sex, SBP, BMI, TC, and the HDL-C level. ^a^The logarithmic transformation was performed to calculate the *P* values. ^b^Modified Box-Cox transformation was applied to calculate *P* values. ^c^Significant difference (P < 0.05, after Bonferroni’s correction for post hoc analysis). Abbreviations: ANCOVA, analysis of covariance; BMI, body mass index; DI, disposition index; FLI, fatty liver index; HDL-C, high-density lipoprotein cholesterol; HOMA-IR, homeostasis model assessment of insulin resistance; ISI, insulin sensitivity index; ISSI-2, insulin secretion-sensitivity index-2; OGTT, oral glucose tolerance test; SBP, systolic blood pressure; TC, total cholesterol.

We used a multiple linear regression analysis to evaluate the association between the FLI, insulin sensitivity, and secretion markers in the NGT state. The standardized β coefficients for the FLI were calculated for each marker, and the results are summarized in Table 2. The FLI was not significantly associated with the oral DI_60_. After adjusting for age, SBP, DBP, TC, and HDL-C, the FLI was positively correlated with the HOMA-IR and negatively correlated with the composite ISI and ISSI-2. However, after further adjustment for BMI, only the composite ISI retained a statistically significant correlation with the FLI (β= −0.024, *P* = 0.036, adjusted R^2^ = 0.105). Additionally, we conducted an analysis of covariance with covariate adjustments to examine the differences across the categorized FLI groups (Fig 2). We selected covariates, including age, sex, SBP, BMI, TC, and HDL-C. The HOMA-IR and composite ISI showed significant differences between the FLI groups (P < 0.0001). The oral DI_60_ and ISSI-2 demonstrated statistically significant differences across the FLI groups, even after covariate adjustment (P = 0.027 and P = 0.011, respectively). A further post hoc analysis indicated that the oral DI_60_ and ISSI-2 were statistically significantly lower in the high FLI group than those of the intermediate FLI group.

**Table 2:** Standardized beta coefficient of the fatty liver index (FLI) for insulin sensitivity or secretion markers according to the multivariate linear regression.

	HOMA-IR^a^		Composite ISI^a^		Oral DI60^b^		ISSI-2^a^	
	β	*P*	β	*P*	β	*P*	β	*P*
Model 1	0.129	<0.0001	−0.162	<0.0001	0.010	0.475	−0.036	<0.0001
Model 2	0.108	<0.0001	−0.125	<0.0001	−0.004	0.816	−0.039	<0.0001
Model 3	−0.008	0.576	−0.024	0.036	0.012	0.571	−0.015	0.142
Adjusted R^2^	0.062		0.105		0.001		0.012	

Independent variable in model 1: the FLI

Independent variables in model 2: the FLI, age, SBP, DBP, TC, and HDL-C

Independent variables in model 3: the independent variables in model 2 plus BMI.

^a^Logarithmic transformation was performed prior to the analysis.

^b^Modified Box-Cox transformation was applied prior to the analysis.

Abbreviations: BMI, body mass index; DBP, diastolic blood pressure; DI, disposition index; FLI, fatty liver index; HDL-C, high-density lipoprotein cholesterol; HOMA-IR, homeostasis model assessment of insulin resistance; ISI, insulin sensitivity index; ISSI-2, insulin secretion-sensitivity index-2; SBP, systolic blood pressure; TC, total cholesterol.

### Risk of the incidence of T2DM across the FLI groups

**Fig 3:**
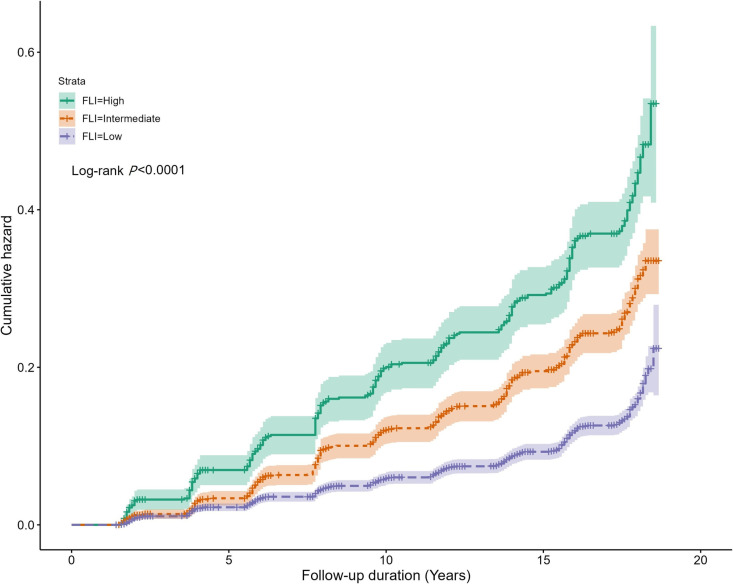
Cumulative incidence of T2DM over the 18-year follow-up period across the FLI groups. Abbreviations: FLI, fatty liver index; T2DM, type 2 diabetes mellitus.

We analyzed the cumulative incidence of T2DM during the 18-year follow-up period. The incidence was calculated biennially. Of the 6,083 participants, 1,006 (16.5%) individuals developed T2DM. In the low FLI group, the cumulative incidence of T2DM was 11.3% (n = 430). The high FLI group comprised 31.9% of the patients (n = 233). The median follow-up duration was 12.4 years (interquartile range, 9.8–17.8 years). The cumulative incidence of T2DM over the course of 18 years was illustrated using a cumulative incidence curve (Fig 3). The high FLI group exhibited a significantly higher incidence rate, as indicated by the curve (log-rank test; P < 0.0001).

We conducted a multivariate Cox proportional hazards regression analysis to determine whether the FLI was an independent predictor of incident T2DM (Table 3). In model 1, the HRs for incident T2DM were 2.08 (95% CI, 1.80–2.40) for the intermediate FLI group and 3.41 (95% CI, 2.91–4.00) for the high FLI group. In model 2, we adjusted for factors known to influence the occurrence of T2DM, such as age, sex, BMI, TC, HDL-C, SBP, current smoking status, alcohol intake, and family history of diabetes [27]. The HRs were 1.71 (95% CI, 1.43–2.05) and 2.57 (95% CI, 2.02–3.26) for the intermediate and high FLI groups, respectively. In model 3, which was further adjusted for insulin sensitivity and secretion markers, including the HOMA-IR, composite ISI, oral DI_60_, and ISSI-2, the HRs were 1.57 (95% CI, 1.31–1.88) for the intermediate FLI group and 1.96 (95% CI, 1.54–2.50) for the high FLI group.

**Table 3:** Hazard ratios and 95% confidence intervals of T2DM according to the FLI.

Variable	FLI		
	Low (<30)	Intermediate (30–59)	High (≥60)
n	3791	1562	730
DM incidence (%)	430 (11.3)	343 (22.0)	233 (31.9)
Median follow-up, years (IQR)	13.9 (11.8, 17.8)	13.2 (9.6, 17.8)	12.2 (6.3, 17.7)
Person-years follow-up	52660.3	20673.7	8880.5
Incidence rate/ 1000 person-years	8.17	16.6	26.2
Model 1	1 (reference)	2.08 (1.8–2.39)	3.42 (2.91–4.00)
Model 2	1 (reference)	1.71 (1.43–2.05)	2.57 (2.02–3.26)
Model 3	1 (reference)	1.57 (1.31–1.88)	1.96 (1.54–2.50)

Model 1: crude

Model 2: adjusted for age, sex, alcohol intake, smoking, BMI, SBP, TC, HDL-C, and family history of diabetes.

Model 3: adjusted for the variables used in model 2 plus the HOMA-IR^a^, composite ISI^a^, IGI_60_^b^, and ISSI-2^a^.

^a^Logarithmic transformation was performed prior to analysis.

^b^Modified Box-Cox transformation was applied prior to analysis.

Abbreviations: BMI, body mass index; FLI, fatty liver index; DM, diabetes mellitus; HOMA-IR, homeostasis model assessment of insulin resistance; IQR, interquartile range; IGI, insulinogenic index; ISI, insulin sensitivity index; ISSI-2, insulin secretion-sensitivity index-2; SBP, systolic blood pressure; TC, total cholesterol.

Additionally, to investigate sex-specific differences in the incidence of T2DM in our study, we conducted a sex-stratified multivariate Cox proportional hazards regression analysis (Table S1). Interaction terms (sex × FLI groups) were not statistically significant in any of the three models (all P for interaction >0.05).

### Predictive ability of the FLI for T2DM

**Fig 4:**
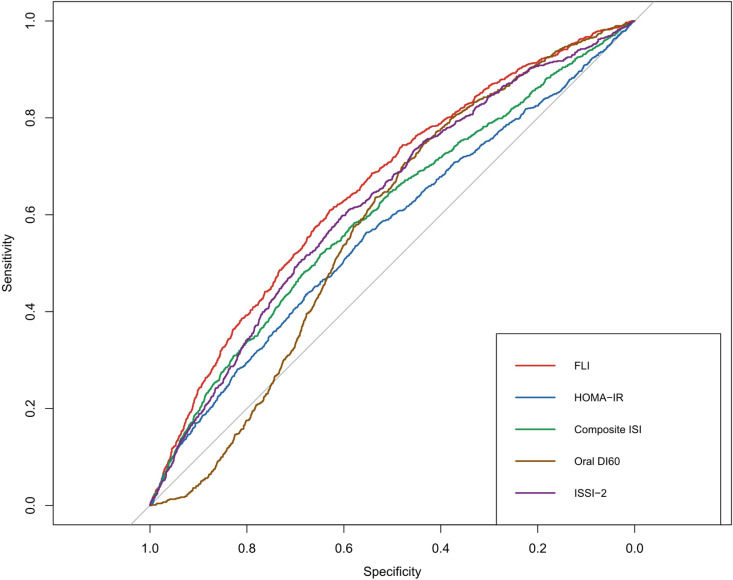
ROC curve of the FLI and insulin sensitivity and secretion markers. Abbreviations: FLI, fatty liver index; ROC, receiver-operating characteristic.

We evaluated the predictive ability of the FLI for T2DM and compared it with that of insulin sensitivity and secretion markers. We conducted a ROC analysis that revealed that the FLI had the highest AUC value at 0.654 (95% CI, 0.636–0.673); ISSI-2, composite ISI, oral DI_60_, and HOMA-IR had the next highest AUC values (Table 4 and Fig 4). Differences between the AUCs were tested using the DeLong test, which showed that the FLI was significantly different from other insulin markers (P < 0.05).

**Table 4:** Predictive ability of the FLI for T2DM.

	AUC (95%CI)	Youden index	*P*
FLI	0.654 (0.636–0.673)	0.239	Reference
ISSI-2^a^	0.625 (0.607–0.644)	0.202	0.0288
Composite ISI^a^	0.601 (0.581–0.620)	0.166	<0.0001
Oral DI_60_^b^	0.575 (0.558–0.593)	0.182	<0.0001
HOMA-IR^a^	0.566 (0.546-0.586)	0.117	<0.0001

^a^Logarithmic transformation was performed prior to the analysis.

^b^Modified Box-Cox transformation was applied prior to the analysis.

AUC, area under the curve; CI, confidence interval; DI, disposition index; FLI, fatty liver index; FPG, fasting plasma glucose; ISI, insulin sensitivity index; ISSI-2, insulin secretion-sensitivity index-2; HOMA-IR, homeostasis model assessment of insulin resistance; T2DM, type 2 diabetes mellitus; TG, triglyceride.

## Discussion

Our large-scale, community-based, retrospective cohort study showed that a high FLI, even for individuals with NGT, could serve as an early predictor of T2DM. High FLI levels are associated with reduced insulin sensitivity and secretion. The FLI exhibited a slightly better predictive ability than other insulin sensitivity and secretion markers. Furthermore, the FLI remained independently associated with the incidence of T2DM, even after adjusting for common metabolic factors related to T2DM development, insulin sensitivity, and secretion markers.

The Diabetes Prevention Program focused on individuals with prediabetes to study the outcomes related to progression to T2DM. Among those who experienced progression to T2DM, clear differences were noted when they were compared with individuals who did not experience progression to T2DM. Those who experienced progression to T2DM exhibited low liver attenuation values (<40 Hounsfield units), indicating a fatty liver, high level of insulin resistance, high WC, and high TG level [28]. These factors correspond to the components included in the FLI. However, the absence of initial data regarding fatty liver from the start of the Diabetes Prevention Program study made it difficult to definitively establish causality. We compared the initial data and data obtained during 18 years of follow-up to determine the prevalence of T2DM among the FLI groups.

Previous FLI studies have primarily focused on populations with prediabetes at high risk for progression to new-onset T2DM. The results indicated that the FLI is a simple and practical tool that can be used to predict progression to T2DM [16,17,29]. Additionally, several studies have investigated the role of the FLI as a predictive factor for the general population, including individuals without diabetes [30–32]. However, those studies only included individuals with prediabetes. Because glucose intolerance increases the risk of T2DM, studies that specifically focused on populations with normal glucose tolerance have been relatively limited. A study in Japan that used real-world data analyzed the incidence of T2DM according to FLI groups in populations with and without IFG. However, individuals with IGT within the prediabetes range were not excluded because OGTT data were not used. Therefore, this population cannot be truly defined as having NGT [33]. Additionally, it is important to note that the study performed in Japan did not compare insulin resistance and was not adjusted for it, which could have influenced the results. The lower incidence of T2DM observed in individuals without IFG in that study compared to that of our study may be attributable to the small sample size and lack of OGTT results, which prevented the complete identification of true diabetes cases. To the best of our knowledge, only one study has included populations with NGT as determined by the OGTT. A study conducted in Spain focused on individuals with NGT and compared populations with NGT and prediabetes separately. Among the 2,260 participants, 641 had prediabetes and 1,619 had OGTT-confirmed NGT. The Cox regression model included the HOMA-IR as an adjustment variable. In the ROC curve analysis, the AUC values were calculated for each group; the addition of the FLI to conventional T2DM risk factors resulted in an improvement in the AUC values. This improvement was particularly evident in models that did not include the HOMA-IR [34]. In our study, among individuals with NGT, the correlation between the FLI and composite ISI was stronger than that between the FLI and HOMA-IR. Additionally, in terms of the predictive value, the FLI had the highest AUC; the ISSI-2 and composite ISI had the next highest AUC. Therefore, as a predictor of T2DM before the onset of glucose intolerance, the FLI appears to be superior to the HOMA-IR and other insulin markers. Notably, our study included a large population and excluded individuals with IFG and IGT based on the OGTT results. Moreover, we compared insulin markers of individuals with OGTT-confirmed NGT and identified their differences. After adjusting for insulin sensitivity markers, we also demonstrated that the FLI differed significantly even among individuals with NGT.

The mechanisms that link NAFLD and T2DM remain unclear. However, lipid accumulation in the liver is linked to both hepatic insulin resistance and hepatic inflammation. Moreover, currently, increasing evidence indicates that lipid accumulation in the liver is also closely associated with insulin resistance in adipose tissue and muscle [35–37]. In our study, individuals with normal glucose levels in the high FLI group exhibited low insulin sensitivity and secretion. A dose-response meta-analysis revealed a direct relationship between the FLI and incidence of T2DM [38]. Therefore, we further aimed to investigate the association between the FLI and established insulin resistance markers of individuals with NGT. As shown in Table 2, the multivariate linear regression analysis of model 1 demonstrated a linear relation among the FLI and HOMA-IR, composite ISI, and ISSI-2. However, the oral DI_60_ did not exhibit a significant linear association. In model 3, the composite ISI remained significantly associated after adjusting for BMI, whereas the associations with HOMA-IR and ISSI-2 disappeared. This suggests that these markers are highly associated with BMI. In contrast, a group-based analysis revealed that the differences between the FLI groups remained statistically significant even after adjusting for BMI (Fig 2). Participants in the high FLI group exhibited more pronounced impairments in insulin secretion. Both the oral DI_60_, which is associated with early insulin secretion, and the ISSI-2, which is an insulin secretion index adjusted for insulin sensitivity, of the high FLI group were lower than those of the intermediate FLI group.

After performing multivariate Cox proportional hazards regression, we demonstrated that the FLI remained independently associated with the incidence of T2DM even after adjusting for established metabolic factors, insulin sensitivity, and secretion markers (Table 3). Additionally, compared with insulin resistance and secretion markers, the FLI had a better predictive rate for T2DM. However, the AUC value was approximately 0.654, which was not particularly high. However, this low predictive rate may have occurred because our study focused on individuals with NGT and included approximately half of the participants in the low FLI group, who likely had a low probability of NAFLD.

This study had several strengths. First, we analyzed data of a large, community-based cohort who underwent extensive follow-up for 18 years and provided robust insights into disease progression. Second, to our knowledge, this is the first study to evaluate the role of the FLI as an early predictor of T2DM in individuals with NGT as determined by OGTT results in Asia. Our findings underscore the potential of the FLI as a noninvasive tool that can be used to assess the early T2DM risk, thus suggesting that liver steatosis may predict T2DM development earlier than previously anticipated. Finally, this is the first study to compare the predictive ability of the FLI with OGTT-derived insulin sensitivity and secretion markers; the results of this comparison demonstrated that the FLI outperformed these markers. Our study had several limitations. First, we excluded data regarding lifestyle factors, particularly physical activity, which is an important factor in the progression of NGT to prediabetes and T2DM. because of the nature of the data, it was challenging to accurately account for physical activity; therefore, it was excluded from the analysis. Second, this cohort study measured data at 0, 60, and 120 min during the OGTT, thus limiting the ability to use blood glucose and insulin values at 30 min. Therefore, OGTT-derived insulin sensitivity and secretion markers based on these measurements may not fully reflect insulin secretion and sensitivity of adults with NGT [39]. However, the oral DI_60_ calculated from the IGI_60_ demonstrated a strong correlation with the 30-min IGI (IGI_30_), making it a valid indicator of early insulin secretion. Additionally, the ISSI-2, as derived from the OGTT, exhibits a moderate correlation with the DI, supporting its reliability [24]. Third, misclassification of the NGT status may occur because of the limited reproducibility of a single OGTT measurement because individual glucose tolerance may exhibit some degree of variability [40]. However, our study included a large-scale cohort and the OGTT was conducted at baseline, which may help mitigate this limitation.

In conclusion, we demonstrated that the FLI can serve as an early predictor of T2DM, even among individuals with NGT. By comparing the FLI with established markers of insulin resistance and insulin secretion, we further evaluated its ability to reflect underlying insulin sensitivity and β-cell function. These findings emphasize the critical role of liver steatosis, as indicated by the FLI, in the early pathogenesis of T2DM, even among individuals with NGT. Therefore, individuals with a high FLI and NGT may be at high risk for metabolic dysfunction with liver steatosis, thus emphasizing the need for early preventive lifestyle modifications to mitigate the development of T2DM.

## Supporting information

S1 Table Hazard ratios and 95% confidence intervals of T2DM according to the FLI, stratified by sex. (DOCX)
