# Supplementary material for: Fatty liver index and risk of type 2 diabetes of adults with normoglycemia: Insights into insulin sensitivity and beta-cell function
Source: PLoS One. 2025 Jun 26;20(6):e0327058. doi: 10.1371/journal.pone.0327058 (PMC12200831; doi:10.1371/journal.pone.0327058)
Supplement: S1 Table — (DOCX) [file pone.0327058.s001.docx]

**Table S1. Hazard ratios and 95% confidence intervals of T2DM according to the FLI, stratified by sex.**

|  | **Men** | | | **Women** | | |
| --- | --- | --- | --- | --- | --- | --- |
|  | **Low (<30)** | **Intermediate**  **(30-59)** | **High (≥60)** | **Low (<30)** | **Intermediate**  **(30-59)** | **High (≥60)** |
| n | 1455 | 906 | 530 | 2336 | 656 | 200 |
| DM incidence (%) | 164 (11.3) | 196 (21.6) | 170 (32.1) | 266 (11.4) | 145 (22.1) | 63 (31.5) |
| Model 1 | 1 | 2.01(1.63-2.47) | 3.30 (2.66-4.09) | 1 | 2.10 (1.72-2.57) | 3.43 (2.60-4.51) |
| Model 2 | 1 | 1.87(1.46-2.40) | 2.95 (2.16-4.03) | 1 | 1.55 (1.19-2.01) | 2.20 (1.49-3.26) |
| Model 3 | 1 | 1.75 (1.36-2.25) | 2.17 (1.58-2.98) | 1 | 1.37 (1.06-1.78) | 1.78 (1.20-2.63) |

Model 1: crude.

Model 2: adjusted for age, sex, alcohol intake, smoking, BMI, SBP, TC, HDL-C, and family history of diabetes.

Model 3: adjusted for the variables used in model 2 plus the HOMA-IR, composite ISI, IGI_60_, and ISSI-2.

*Interaction terms (sex×FLI groups) were not statistically significant in any of the three models (all P for interaction > 0.05), suggesting that the association between the FLI stage and diabetes risk did not differ by sex.

^a^Logarithmic transformation was performed prior to analysis.

^b^Modified Box-Cox transformation was applied prior to analysis.

Abbreviations: BMI, body mass index; DM, diabetes mellitus; HDL-C, high-density lipoprotein cholesterol; HOMA-IR, homeostasis model assessment of insulin resistance; ISI, insulin sensitivity index; IGI_,_ insulinogenic index; ISSI-2, insulin secretion-sensitivity index-2; SBP, systolic blood pressure; TC, total cholesterol.
